# A prospective evaluation of the contribution of ambient temperatures and transport times on infrared thermometry readings of intravenous fluids utilized in EMS patients

**DOI:** 10.1186/s12245-014-0047-y

**Published:** 2014-12-16

**Authors:** Jeremy Joslin, Andrew Fisher, Susan Wojcik, Derek R Cooney

**Affiliations:** Department of Emergency Medicine, State University of New York Upstate Medical University, Syracuse, NY 13210 USA; State University of New York Upstate Medical University, Syracuse, NY 13210 USA; EMSTAT Center, 550 East Genesee St., Syracuse, NY 13202 USA

**Keywords:** Ambulance, Emergency department, Emergency medical services, Environmental medicine, Fluid, Hypothermia, Intravenous, Saline, Temperature

## Abstract

**Background:**

During cold weather months in much of the country, the temperatures in which prehospital care is delivered creates the potential for inadvertently cool intravenous fluids to be administered to patients during their transport and care by emergency medical services (EMS). There is some potential for patient harm from unintentional infusion of cool intravenous fluids. Prehospital providers in these cold weather environments are likely using fluids that are well below room temperature when prehospital intravenous fluid (IVF) warming techniques are not being employed. It was hypothesized that cold ambient temperatures during winter months in the study location would lead to the inadvertent infusion of cold intravenous fluids during prehospital patient care.

**Methods:**

Trained student research assistants obtained three sequential temperature measurements using an infrared thermometer in a convenience sample of intravenous fluid bags connected to patients arriving via EMS during two consecutive winter seasons (2011 to 2013) at our receiving hospital in Syracuse, New York. Intravenous fluids contained in anything other than a standard polyvinyl chloride bag were not measured and were not included in the study. Outdoor temperature was collected by referencing National Weather Service online data at the time of arrival. Official transport times from the scene to the emergency department (ED) and other demographic data was collected from the EMS provider or their patient care record at the time of EMS interaction.

**Results:**

Twenty-three intravenous fluid bag temperatures were collected and analyzed. Outdoor temperature was significantly related to the temperature of the intravenous fluid being administered, *b* = 0.69, *t*(21) = 4.3, *p* < 0.001. Transport time did not predict the measured intravenous fluid temperatures, *b* = 0.12, *t*(20) = 0.55, *p* < 0.6.

**Conclusions:**

Use of unwarmed intravenous fluid in the prehospital environment during times of cold ambient temperatures can lead to the infusion of cool intravenous fluid and may result in harm to patients. Short transport times do not limit this risk. Emergency departments should not rely on EMS agencies’ use of intravenous fluid warming techniques and should consider replacing EMS intravenous fluids upon ED arrival to ensure patient safety.

## Background

Many urban and rural emergency medical service (EMS) systems experience freezing temperatures on a regular basis. The United States National Oceanic and Atmospheric Administration (NOAA) National Climatic Data Center reports with 90% probability that our receiving hospital located in Syracuse, New York (NY) experiences a temperature at or below freezing 215 days per year and more generalizable, the entire Northeast, most of the Midwest, and the Great Plains north of Nebraska all experience similar temperature patterns with regard to number of days below freezing [1]. This preponderance of cold temperatures in which prehospital care is delivered creates the potential for cool intravenous (IV) fluids to be administered to patients during their transport and care.

Gentilello has equated the effect of unwarmed intravenous fluids (IVFs) on body temperature to that of a patient who must use 16 kcal of energy to warm a liter of room temperature (21°C) crystalloid to 37°C. If a 70-kg patient is unable to generate that heat, a decrease in body temperature of 0.3°C occurs which is enough to initiate shivering [2]. While prospective data conflict on the relationship between mortality and the presence of hypothermia in trauma patients, complications associated with hypothermia and morbidity have been associated [3,4].

Intravenous fluid warmers are abundant in the commercial market, and ‘homemade’ methods of warming IVFs abound [5,6]. Still, local use of these warmers seems non-existent. We sought to determine whether winter temperatures realistically affect the temperature of IVFs stored on an ambulance and whether short transport times mitigated the need for any warming devices.

## Methods

We prospectively measured the temperature of intravenous fluids being administered to patients arriving to our emergency department by EMS. Trained student research assistants obtained a convenience sample of observations during two consecutive winter seasons (2011 to 2013) at our receiving hospital in Syracuse, New York (NY).

Temperatures of the intravenous fluid bags were measured as soon as possible after entering the emergency department. Three sequential temperature measurements were taken by aiming an infrared laser thermometer (Black & Decker TLD100 Thermal Leak Detector, Black & Decker, Inc, New Britain, CT) approximately 2 to 3 cm away from the fluid’s bag. During data organization, the average of these three measurements was calculated. Intravenous fluids contained in anything other than a standard polyvinyl chloride bag were not measured and were not included in the study.

Outdoor temperature was collected by referencing National Weather Service online data (http://www.weather.gov) for our zip code (13210) at the time of arrival using a hospital computer. Official transport times from the scene to the emergency department and other demographic data were collected from the EMS provider or their patient care record at the time of EMS interaction.

Linear regression analysis of outdoor temperature, IVF temperature on arrival, and transportation times was performed using SPSS v 19.0®. Review by our Institutional Review Board determined that our research protocol was ‘not human subjects research.’

## Results

The temperature data from 23 patients presenting by EMS were obtained and analyzed (*N* = 23). One patient’s transport time was not recorded but temperature data was used in analysis. Table 1 describes characteristics of measurements obtained.

**Table 1:** **Characteristics of temperatures and transport times measured**

	**Maximum**	**Minimum**	**Median**	**Percentiles**
Outdoor temperatures (°C)	14	−11	−3.0	25% −6
				50% −3
				75% 0
IV fluid temperatures (°C)	31.3	17.6	20.2	25% 18.9
				50% 20.2
				75% 22.2
Transport times (min)	81	7	13.5	25% 10.5
				50% 13.5
				75% 40.0

**Figure 1:**
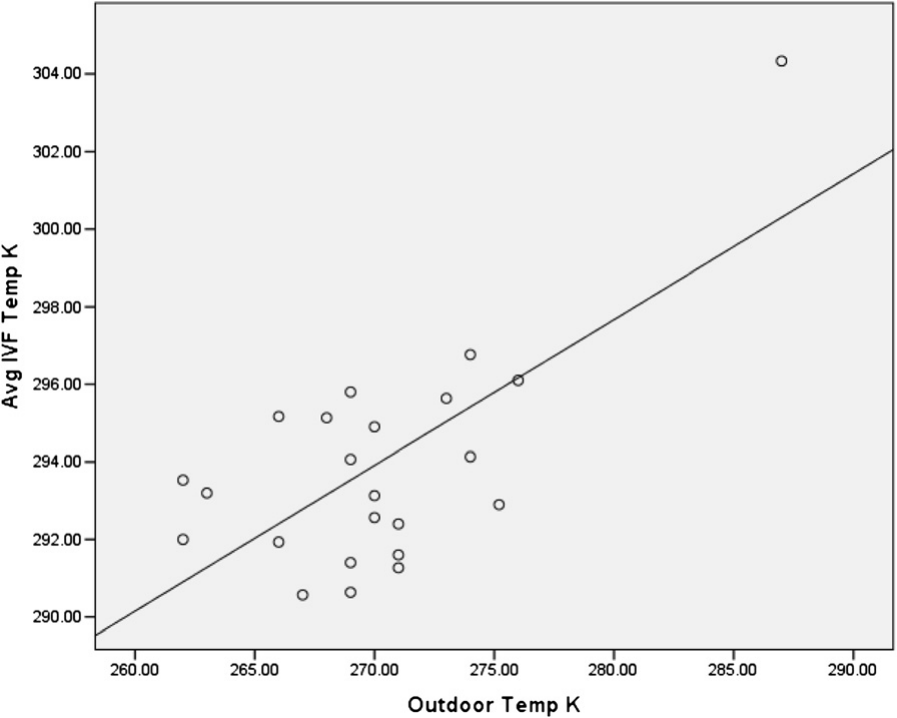
**Scatter plot of outdoor temperatures against IV fluid temperatures measured.**

The outdoor temperature measured significantly predicted the temperature of the IVF being administered, *b* = 0.69, *t*(21) = 4.3, *p* < 0.001. The outdoor temperature measured also explained a significant proportion of variance in the measured temperature of the IVF, R2 = 0.47, *F*(1, 21) = 18.64, *p* < 0.001. Conversely, the transport time did not predict the measured IVF temperatures, *b* = 0.12, *t*(20) = .55, *p* < 0.6. Figure 1 illustrates results on a scatter plot.

## Discussion

Although prospective trials conflict on the effect of hypothermia on mortality, some effect on morbidity have been shown [3,4]. Hypothermia can potentially lead to life threatening complications including increased cardiac oxygen demand, cardiac depression, respiratory depression, ileus, hyperglycemia, altered mental status, platelet dysfunction, and coagulation and fibrinolytic dysfunction [5]. Within our EMS region, administration of unwarmed IVFs to patients appears to be a common practice during cold, winter months. Our data demonstrate a logical relationship between ambient outdoor temperature and the temperature of crystalloid fluid being delivered intravenously and are consistent with previous data collection [7]. It is certainly understandable that the colder the weather is, the greater the chance that bags of IVFs sitting in an ambulance will be cooled.

Our data also show that transport time cannot be used to predict the temperature of IVF despite an inclination that short transport times might be protective of the fluid bag temperatures. We believe this data uncovers a possible misperception by transporting EMS agencies: specifically, that warming of their IVF stock is unnecessary within a small coverage area with relatively short transport times. On the contrary, we believe the data suggest that all EMS agencies who operate in cold climates would likely benefit from a methodology to keep intravenous fluids warm while they are stocked on an ambulance. It is likely that the temperature of the IVF is more a function of storage time than of administration time. Receiving emergency departments should be aware of this phenomenon.

Lyng et al. demonstrated that liter bags of IVF could be warmed by the dashboard heater of an ambulance *en route* to a call [8]. While this approach is not specifically advocated, it does appear that alternatives to commercial devices may be equally effective. Cooling of the IVF during the transit from the bag, through tubing, and into a catheter is another issue altogether with similar hurdles and considerations [9]. Emergency department staff should understand the local practices of the EMS transporting services and should be aware of improvisation methods which may or may not be utilized.

We believe that the strength of prediction between outdoor temperature and IVF temperature may have been stronger with a larger dataset. In our sample, one fluid bag was measured at 31.3°C which was considerably warmer than ambient temperature outside at that time (14°C). It is likely that this patient’s fluid had been warmed before or during transport, yet our study was not designed to capture these details and confounders. Future investigations should focus on EMS compliance with best practices of delivering warmed fluids to patients in cold winter environments in order to inform the practice of emergency nurses receiving these patients.

### Limitations

This study is limited by the nature of convenience sampling. Our research assistants’ schedule was managed independent of the needs of this particular study; however, it is unlikely that more structured sampling would affect our results. Although other methods of measuring the IVF more directly are possible, we feel that our measurements were likely accurate. Indeed, infrared thermometry as employed in this investigation has been shown to be accurate for measuring temperatures of intravenous crystalloid fluid [10].

In the interest of patient safety, it may be appropriate for emergency departments to consider polices that require the replacement of EMS intravenous fluids with hospital intravenous fluids that have been stored at temperatures closer to normal body temperature. In addition, these policies should take into consideration the amount of fluid administered by EMS as additional patient warming may be clinically indicated.

## Conclusions

The lack of EMS agencies’ use of intravenous fluid warming devices during winter months could contribute to mild hypothermia and possibly portend a deleterious effect on health. Even short transit times do not seem to protect against this risk. Use of IVF warming devices by EMS may not be routine.

## Abbreviations

ED: Emergency department
EMS: Emergency medical services
IV: Intravenous
IVF: Intravenous fluid
NOAA: National Oceanic and Atmospheric Administration
